# Poor mental health and adverse psychosocial conditions among adolescents in the Nordic countries: contrasting variable- and person-centered analyses

**DOI:** 10.3389/fpubh.2025.1604785

**Published:** 2025-05-16

**Authors:** Håkan Stattin, Charli Eriksson, Einar Baldvin Thorsteinsson

**Affiliations:** ^1^Department of Psychology, Uppsala University, Uppsala, Sweden; ^2^Department of Learning, Informatics, Management and Ethics, Karolinska Institute, Stockholm, Sweden; ^3^Faculty of Medicine and Health, School of Psychology, University of New England, Armidale, NSW, Australia

**Keywords:** adolescents, mental health, health profiles, psychosomatic complaints, self-rated health, life satisfaction, sex differences, variable- and person-centered analyses

## Abstract

**Background:**

The aim is to compare conclusions about the role of adolescent mental health in adverse psychosocial conditions depending on the analytic approach used (variable-centered vs. person-centered). In the variable-centered analyses, three mental health indicators (psychosomatic complaints, self-rated poor health, and low life satisfaction) were examined in relation to adverse physical, school, interpersonal, and personal conditions. In the person-centered analyses, the three health indicators were used to create mental health profiles using cluster analysis, which were examined in relation to the adverse psychosocial measures.

**Methods:**

Data were drawn from the HBSC survey of 15-year-olds in 2022. Samples from five Nordic countries were used (*N* = 7,860). Correlational and cluster analysis were applied.

**Results:**

The variable-oriented analyses show that all three health indicators were interrelated (ranging between 0.45 and 0.53), form one common factor (with factor loadings ranging from 0.66 to 0.78), and that psychosocial problems do not stand out as having different associations from the other two indicators. Cluster analysis of the three health indicators revealed seven health profiles. These profiles were differentially associated with the outcome measures examined. The health profiles associated with lack of physical activity all included self-rated poor health. The health profiles associated with adverse school, interpersonal, and personal conditions all included high psychosomatic complaints. As a proxy for mental health, psychosomatic complaints have been the primary measure in many previous studies when associated with aversive psychosocial conditions. However, the mental health profile characterized by high levels of psychosomatic complaints exclusively had average levels of these adverse psychosocial measures.

**Conclusion:**

Variable- and person-centered approaches to the study of adolescent mental health provide complementary insights into the role of the three health indicators in relation to adverse psychosocial conditions. The person-centered approach provides much needed additional information about when a specific health indicator is associated with adverse psychosocial conditions and when it is not. As such, person-centered analyses are needed for future studies in other domains that wish to tell a more complete story as part of their findings.

## Introduction

The Health Behaviour in School-aged Children (HBSC) is a collaborative, cross-national initiative that monitor many aspects of health and adjustment in nationally representative samples of school children every four years. Over the years, HBSC studies have measured somatic and psychological complaints and life satisfaction as indicators of subjective experiences of mental health (1–3). Adolescents’ perceptions of their overall health, whether physical or psychological, have been used as an additional measure of self-rated health (4, 5). In the present study, we examine how these three health indicators relate to adverse physical, school, interpersonal, and personal conditions in samples from five Nordic countries. We compare information from variable- and person-centered analyses.

### Background

Decades of research have considered psychosomatic complaints as the prime candidate for broader mental health problems, mainly using variable-centered methods to relate these complaints to various measures of individual, developmental, social, economic, and cultural conditions. Life satisfaction has also been widely used as a measure of mental health, often in combination with psychosomatic complaints (2). In addition, there are good reasons to believe that self-rated health should be considered as an additional indicator of adolescents’ health status.

First, existing longitudinal analyses suggest that psychosomatic complaints and self-rated health lead to both similar and different future outcomes. Psychosomatic complaints are primarily associated with future anxiety and depression (6–11), whereas self-rated poor health is mainly associated with future morbidity and mortality (12, 13). Both psychosomatic complaints and self-rated poor health are associated with medication use and use of medical services (14, 15). The conclusion that can be drawn from these longitudinal analyses is that to gain a comprehensive understanding of adolescent health as a risk factor for future health problems, studies should simultaneously track risk conditions among adolescents for both psychosomatic complaints and self-rated health.

A paucity of information exists regarding the prospective implications of low life satisfaction, with the majority of extant studies employing life satisfaction as an outcome measure. A multitude of longitudinal studies have examined a variety of potential explanations for changes in life satisfaction. In contrast to research on psychosomatic complaints and self-rated poor health, the results of studies using life satisfaction as a predictor variable are not consistent [e.g., (16–21)]. However, to comprehensively capture the spectrum of health challenges faced by adolescents, it seems imperative to elucidate the interplay among these three health indicators: psychosomatic complaints, self-rated health, and life satisfaction.

Second, psychosomatic complaints, self-rated health, and life satisfaction have all been used as indicators of adolescents’ conception of their broader mental wellbeing (22). A key question is whether psychosomatic problems, self-rated poor health, and lack of life satisfaction are all roughly equally associated with measures of adverse psychosocial conditions, or whether one of the three indicators of poor health is more strongly associated with these measures of adverse psychosocial conditions than the others. Because the three indicators are roughly equally related to each other, we will examine if they are roughly equally associated with adverse psychosocial conditions.

Thirdly, there is a compelling rationale for examining these three health indicators in conjunction with each other, thereby incorporating the issue of methodology. Eriksson and Stattin (23) adopted a person-centered approach to examining health among Nordic adolescents. The application of cluster analysis to the data set of psychosomatic complaints and self-rated poor health yielded a fairly consistent cluster solution for all five Nordic countries: Denmark, Finland, Iceland, Norway, and Sweden. The solution consisted of five clusters: “adequate health,” “perceived good health,” “perceived poor health,” “high psychosomatic complaints,” and “dual health problems” (both high psychosomatic problems and self-rated poor health). The observed increase in psychosomatic problems over the past decades has been interpreted as an indication of a broader trend towards increased mental health problems, especially among girls. Indeed, as indicated by the Eriksson and Stattin study, the proportion of respondents in the cluster profiles “high psychosomatic complaints” and “dual health problems” increased more than twofold between 2002 and 2022. This increase was particularly pronounced among girls. However, analyses conducted in 2022 revealed that adolescents in the cluster with dual health problems reported considerably more severe psychosocial problems than those in the other four cluster profiles. The group of adolescents with elevated psychosomatic complaints only exhibited levels of psychosocial challenges that were commensurate with those observed among respondents belonging to the clusters with a perceived poor health status. These findings underscore the importance of considering health indicators in relation to each other and of not regarding adolescents with high psychosomatic symptoms as a homogeneous group.

### Current study

The use of person-centered methods in research has enabled researchers to uncover new understandings of developmental processes (24–26). For example, person-centered approaches have been observed to provide more nuanced insights into interpersonal relations than variable-centered methods (27). Thus, in the present study we will use variable-centered methods (correlations and factor analysis) together with person-centered methods (cluster analysis) to understand how the three health indicators and the resulting health profiles are associated with a battery of outcome measures that tap into adverse physical, school, interpersonal, and personal conditions among adolescents.

## Materials and methods

### Participants

The HBSC Study is an international, collaborative, cross-national survey with the overall goal of improving understanding of the social context, health, and health behaviors of young people aged 11, 13, and 15 years. It is conducted every 4 years. The present study examines data on 15-year-olds in the Nordic countries from surveys in 2022. The number of young people with complete data for all three indicators of poor health was 1,611 in Denmark, 980 in Finland, 2,887 in Iceland, 971 in Norway, and 1,411 in Sweden. In Denmark, Finland, Norway, and Sweden, samples (school classes) were drawn randomly, and stratification was proportional to obtain nationally representative data sets. In Iceland, all schools were invited to participate. Data were collected using self-administered, internationally standardized questionnaires during school hours after instruction by the teacher. Students were informed verbally and in writing of the confidentiality of their responses, and participation was confidential and voluntary.

A standardized international research protocol was followed to ensure consistency in survey instruments and data collection and processing procedures. Schools or classes that refused to participate and students who were absent on the day of the survey were the main sources of non-response and were not followed up. The HBSC Data Management Centre at the University of Bergen, Norway, checked the quality of the data collected, performed appropriate cleaning of the data and merged the national data sets into a Nordic data file. The methodology for data collection is described in the HBSC protocol (28), which requires consistency in sampling plans, survey instruments, and data collection.

### Measures

The indicators of poor health used in the cluster analyses, namely psychosomatic complaints, self-rated poor health, and low life satisfaction, are presented in Table 1. In addition, as reported in the table, a wide range of measures of adverse physical, school, interpersonal and personal conditions are employed.

**Table 1:** Scales and items used in the study.

Concept	Items and alpha reliability	Response scale
*Psychosomatic complaints (HBSC-SCL)*		
“In the last 6 months, how often have you had the following…” and the eight items: ‘headache’, ‘stomachache’, ‘backache’, ‘feeling low’, ‘irritability or bad temper’, ‘feeling nervous’, ‘difficulty falling asleep’ and ‘feeling dizzy’.	8/0.86	1 (*about every day*) to 5 (*rarely or never*) reversely coded
*Self-rated poor health*		
“Would you say your health is …?”	1	1 (*excellent*) to 4 (*poor*)
*Low life satisfaction*		
Life satisfaction was a single item, the Cantril Ladder	1	0 (*worst possible life*) to 10 (*best possible life*). reversely coded
*Physical inactivity and sedentary behavior*		
“Over the past 7 days, on how many days were you physically active for a total of at least 60 min per day?”	1	1 (*0 days*) to 8 (*7 days*) reversely coded
“In your free time: which of the following best describes your typical sedentary habits” Only available for Norway and Sweden.	1	1 (*I spend almost none of my free time sitting*) to 5 (*I spend almost all my free time sitting*)
*Negative school experiences*		
“How do you feel about school at present?”	1	1 (*I like it a lot*) to 4 (*I do not like it at all*)
“How pressured do you feel by the schoolwork you have to do?”	1	1 (*not at all*) to 4 (*a lot*)
*Poor parental communication*		
Not talking to parents: “How easy is it for you to talk to the following people about things that really bother you? Mother and father, respectively. Adolescents who answered that they had no contact with the specific parent were assigned a missing value. Correlation between items is 0.62	2	1 (*very easy*) to 4 (*very difficult*)
*Lack of parental support*		
Lack parental support: “Family tries to help,” “Emotional help from family,” “Talk about problems with family,” “Family help with decisions”	4/0.95	1 (*very strongly disagree*) to 7 (*very strongly agree*) reversely coded
*Lack of teacher and classmate support*		
Lack teachers’ support: “Teachers accept me,” “Teachers care about me,” “I feel trust in my teachers”	3/0.89	1 (*strongly agree*) to 5 (*strongly disagree*)
Lack classmates’ support: “Students like being together,” “Students kind and helpful,” “Students accept me”	3/0.85	1 (*strongly agree*) to 5 (*strongly disagree*)
*Adverse personal conditions*		
Feeling stressed: Two items from Cohen’s Perceived Stress Scale (PSS-10) “Felt unable to control important things in life,” “Felt difficulties were piling up so high that you could not overcome them.” Correlation between items is 0.53.	2	1 (*never*) to 5 (*always*)
Negative wellbeing: WHO-5 Well-Being Index. Please indicate for each of the five statements which is closest to how you have been feeling over the last two weeks. For example “I have felt cheerful and in good spirits”	5/0.69	1 (*at no time*) to 6 (*all the time*) reversely coded
Feeling lonely: “During the past 12 months, how often have you felt lonely?.”	1	1 (*never*) to 5 (*always*)
Low self-efficacy: Based on Schwartzer’s theoretical contribution “How often do you find solutions for problems, if you try hard enough?,” “How often do you manage to do things you decide to do?.” Correlation between items is 0.64	2	1 (*never*) to 5 (*always*) reversely coded
Low SES: A single question about if family is well off. Not available for Denmark.	1	1 (*family is very well of*) to 5 (*not at all well off*)

### Statistical analyses

Statistical analyses were performed using IBM SPSS (version 30.0). We first examined the relationships between the three indicators of poor health and measures of adverse physical, school, interpersonal, and personal conditions. Next, a factor analysis was performed (principal axis factoring with promax rotation) to examine if the three health indicators formed a single factor. After these variable-centered analyses, cluster analysis was used to identify the existing profiles of psychosomatic complaints, self-rated poor health and low life satisfaction in the five Nordic samples. All three indicators were standardized, and hierarchical cluster analysis (Ward’s method) was used to determine the number of clusters. We set the lower explanatory limit at 67% of the total error sum of squares for the number of clusters selected (26). Knowing the number of clusters, a non-hierarchical cluster analysis, K-means clustering, was then used to arrive at the final cluster solution, following the recommendations of Kinder and colleagues (29).

The adjusted standardized residuals were used in cross-tabulations to estimate differences in the proportions of each cluster between boys and girls and between countries. The adjusted standardized residuals in a contingency table can be roughly interpreted as standard normally distributed. Values greater than or equal to 3.29 or less than or equal to -3.29 indicate that the cell deviates significantly from the null hypothesis at the 0.001 level. Finally, the cluster profiles were compared with one-way ANOVAs on the measures of adverse physical, school, interpersonal, and personal conditions.

## Results

### Variable-centered analyses

Across the five countries the correlations between the measures of psychosomatic complaints, self-rated poor health and low life satisfaction were substantial (between psychosomatic complaints and self-rated poor health 0.45, between psychosomatic complaints and low life satisfaction 0.53 and between self-rated poor health and low life satisfaction 0.51). A factor analysis across countries showed that the three health indicators formed a single factor with factor loadings ranging from 0.66 to 0.78, suggesting that they are measuring a similar underlying construct.

Table 2 shows the correlations between each of these indicators of unhealthiness and the factor score and the different measures of adverse psychosocial conditions. The strongest associations were observed between the three indicators and adverse personal conditions, including stress, negative wellbeing, feelings of loneliness, and low self-efficacy. The mean correlation between psychosomatic complaints and all measures of adverse psychosocial conditions presented in Table 2 was 0.36. The same correlation was observed for low life satisfaction (*r* = 0.37) and somewhat lower for self-rated poor health (*r* = 0.32). Thus, the three health indicators are fairly equally associated with the various adverse psychosocial conditions. Based on these correlations, it would be wrong to conclude that psychosomatic complaints are the specific indicator of exposure or contributor to adverse psychosocial conditions. The poor health factor score, which includes the three indicators, had an average correlation of 0.43 with the measures of adverse psychosocial conditions. This is essentially the information about the three indicators of poor health that correlational analyses can reveal.

**Table 2:** Correlations between three indicators of poor health and a composite measure of poor health status, on the one hand, and measures of adverse physical, school, interpersonal, and personal conditions, on the other.

Measures	Psychosomatic complaints	Self-rated poor health	Low life satisfaction	Poor health status factor score
*Physical inactivity and sedentary behavior*:				
Physical inactivity	0.12	0.18	0.25	0.22
Sedentary behavior	0.21	0.24	0.35	0.31
*Negative school experiences*:				
Not liking school	0.36	0.42	0.29	0.45
Schoolwork pressure	0.41	0.28	0.22	0.37
*Negative interpersonal relations*:				
Not talk to parents	0.34	0.38	0.34	0.43
Lack parental support	0.30	0.33	0.27	0.37
Lack teachers’ support	0.32	0.34	0.25	0.38
Lack classmates’ support	0.33	0.33	0.28	0.38
*Negative personal conditions*:				
Feeling stressed	0.48	0.44	0.36	0.52
Negative wellbeing	0.57	0.62	0.51	0.70
Feeling lonely	0.52	0.50	0.38	0.58
Low self-efficacy	0.32	0.34	0.32	0.40
Mean correlation	**0.36**	**0.37**	0.32	0.43

All correlations are significant at the 0.001 level.

### Person-centered analyses

Having shown that the three indicators of poor health are highly related to each other and are associated with adverse psychosocial conditions to about a similar extent, we moved to person-centered analyses to examine groups of adolescents who are homogeneous with respect to the levels of the three indicators of poor health. Six clusters, accounting for 69.1% of the sums of squares satisfied the lower explanatory limit of number of clusters. Because we were interested in differentiating various combinations of poor health status, we decided to extract a seven-cluster solution rather than a six-cluster solution. The six and the seven cluster solutions yielded almost identical results when comparing between sexes and countries. The seven-cluster solution accounted for 72.1% of the sums of squares. Table 3 shows this cluster solution for the three indicators of poor health.

**Table 3:** Cluster analysis of mental health profiles across the five Nordic countries and sex differences.

Sex	Good overall health	Lower level of complaints	Complaints only	Complaints and low life satisfaction	Complaints and self-rated poor health	Self-rated poor health only	Poor overall health status
Psychosomatic complaints	−0.82	−0.65	0.84	0.75	1.30	−0.22	1.49
Low life satisfaction	−0.73	−0.33	−0.32	1.09	0.52	0.28	2.27
Self-rated poor health	−1.20	0.14	−0.20	−0.08	1.61	1.54	1.79
N	1955	2,185	1,218	1,062	509	494	437
%	24.9	27.8	15.5	13.5	6.5	6.3	5.6
Boys %	71.5^h^	59.8^h^	31.3^l^	32.4^l^	23.0^l^	52.6	27.6^l^
Girls %	28.5^l^	40.2^l^	68.7^h^	67.6^h^	77.0^h^	47.4	72.4^h^

The following guidelines were used for interpreting the clusters: a low standardized centroid value is < −0.70, an average value is between −0.70 and 0.70, and a high value is >0.70. Sex differences: F(6, 7,860) = 955.14, *p* < 0.001, eta^2^ = 0.35. Chi-square analysis adjusted standardized residuals = ^h^ High; ^l^ Low. Eta^2^ as an effect size: 0.01 is a small effect; 0.06 is a medium effect; 0.14 is a large effect.

The first two clusters reflect good health and include 52.7% of all respondents: “Good overall health” and “Low level of psychosomatic complaints.” The other five clusters capture different constellations of health problems: “Psychosomatic complaints only,” “Psychosomatic complaints and low life satisfaction,” “Psychosomatic complaints and self-rated poor health,” “Self-rated poor health only,” and “Poor overall health” (high levels of all three indicators of poor health). Among the clusters, boys were overrepresented in the two clusters indicating good health, and girls were overrepresented in all clusters with high levels of psychosomatic complaints. According to eta^2^, the sex differences yielded a high effect size. There were about equal proportions of boys and girls in the clusters “Self-rated poor health only.”

We examined whether the proportions for the health profiles differed between the five Nordic countries. As shown in Table 4, some differences were observed (especially for the Swedish sample). The crosstabulation between cluster profiles and countries was statistically significant, χ^2^(24, *N* = 7,860) = 367.84, *p* < 0.001, but Cramer’s V is 0.11, which is a small effect size. This observation was consistent in separate analyses for boys (Cramer’s V = 0.10) and girls (Cramer’s V = 0.13). Thus, the proportions of the seven health profiles showed little variation among respondents in the five countries.

**Table 4:** The percentage of respondents in each of the five Nordic countries who belonged to the seven cluster profiles.

Country	Good overall health	Lower level of complaints	Complaints only	Complaints and low life satisfaction	Complaints and self-rated poor health	Self-rated poor health only	Poor overall health status
Denmark	26.8	34.3^h^	9.1^l^	11.0^l^	5.5	7.9	5.3
Finland	19.6^l^	33.6^h^	18.2^h^	8.6^l^	7.9	7.2	5.0
Iceland	25.1	24.8^l^	17.8	11.9	8.2^h^	6.3	6.0
Norway	29.5^h^	27.8	12.2	13.1	4.3	8.0	5.1
Sweden	22.7	22.5^l^	18.6^h^	23.4^h^	4.6	2.6^l^	5.6

Cramer’s V as an effect size: 0.02 is a small effect; 0.20 to 0.60 is a medium effect; >0.60 is a large effect. Chi-square analysis adjusted standardized residuals = ^h^ High; ^l^ Low (3.29 and −3.29). Total sample: χ^2^(24, *N* = 7,860) = 367.84, *p* < 0.001, Cramer’s V = 0.11.

### Health profiles and adverse psychosocial conditions

Table 5 shows how the seven health profiles differ on adverse physical, school, interpersonal, and personal conditions. As might be expected, the outcome was more favorable for respondents in the two clusters reflecting good health than for the participants in the five clusters reflecting various constellations of poorer health. For the latter five clusters, a general rule of interpretation seems to be that health profiles that include only psychosomatic complaints or only self-rated poor health show more favorable outcomes than those health profiles that include two or more of the indicators of poor health. The worst outcomes for poor school experience, negative interpersonal relationships, and negative personal conditions are found among the 5.6% of young people in the “Poor overall health” profile. They differ significantly from the other health profiles on all measures of school, interpersonal and personal conditions.

**Table 5:** Differences in adverse psychosocial conditions between the seven mental health profiles.

Adverse conditions	Good overall health	Lower level of complaints	Complaints only	Complaints and low life satisfaction	Complaints and self-rated poor health	Self-rated poor health only	Poor overall health status
*Physical inactivity and sedentary behavior*:							
Physical inactivity	−0.48^e^	0.05^cd^	−0.04^d^	0.08^c^	0.56^ab^	0.47^b^	0.65^a^
Sedentary behavior	−0.38^c^	0.10^b^	0.06^b^	0.03^b^	0.30^a^	0.42^a^	0.46^a^
*Negative school experiences*:							
Not liking school	−0.45^f^	−0.19^e^	0.02^d^	0.49^b^	0.47^b^	0.12^c^	1.05^a^
School pressure	−0.38^f^	−0.25^e^	0.30^c^	0.36^c^	0.50^b^	−0.03^d^	0.75^a^
*Negative interpersonal relations*:							
Not talking to parents	−0.50^f^	−0.14^e^	0.03^d^	0.38^c^	0.59^b^	0.33^c^	0.94^a^
Lack parental support	−0.34^f^	−0.20^e^	0.01^d^	0.30^c^	0.52^b^	0.21^c^	0.92^a^
Lack teachers’ support	−0.39^e^	−0.17^d^	0.09^c^	0.35^b^	0.39^b^	0.17^c^	0.87^a^
Lack classmates’ support	−0.45^f^	−0.14^e^	0.12^d^	0.34^c^	0.43^b^	0.20^d^	0.83^a^
*Adverse personal conditions*:							
Stress	−0.56^f^	−0.28^e^	0.20^d^	0.51^c^	0.75^b^	0.17^d^	1.05^a^
Negative wellbeing	−0.77^g^	−0.27^f^	0.11^e^	0.62^c^	0.87^b^	0.38^d^	1.52^a^
Feeling lonely	−0.61^f^	−0.27^e^	0.19^d^	0.58^c^	0.71^b^	0.12^d^	1.24^a^
Low self-efficacy	−0.44^f^	−0.11^e^	0.02^d^	0.23^c^	0.53^b^	0.28^cd^	1.00^a^
*Low SES:*							
Family not well off	−0.38^e^	−0.03^d^	−0.06^d^	0.23^c^	0.45^b^	0.27^c^	0.58^a^

The measures of adverse conditions are transformed to Z-scores.

There are significant differences, at the 0.001 level, in all comparisons. Hence, eta^2^, as a measure of effect size, is reported below. Interpret eta^2^ as an effect size: 0.01 is a small effect; 0.06 is a medium effect; 0.14 is a large effect. Different superscripts represent significant differences (*p* < 0.05) between the seven cluster groups employing Student Neuman Keul’s post-hoc test.

Physical inactivity: F(6, 7,795) = 16,946, eta^2^ = 0.12; Sedentary behavior: F(6, 2,262) = 25.77, eta^2^ = 0.07; Not liking school: F (6, 7,672) = 259.06, eta^2^ = 0.17; School pressure: F(6, 7,661) = 196.20, eta^2^ = 0.03; Not talk to parents: F(6, 7,445) = 243.72, eta^2^ = 0.16; Lack parental support: F (6, 7,445) = 167.19, eta^2^ = 0.12; Lack teachers’ support: F(6, 7,771) = 165.8, eta^2^ = 0.12; Lack classmates’ support: F(6, 7,637) = 181.33, eta^2^ = 0.12; Stress: F (6, 7,722) = 407.52, eta^2^ = 0.24; Negative wellbeing: F(6, 7,771) = 889.65, eta^2^ = 0.41; Feeling lonely: F(6, 7,766) = 506.11, eta^2^ = 0.28; Low self-efficacy: F (6, 7,635) = 203.31, eta^2^ = 0.14; Family not well off: F(6, 6,029) = 86.58, eta^2^ = 0.08.

*Physical inactivity and sedentary time.* The three profiles indicating high levels of self-rated poor health were systematically associated with low levels of physical activity and with respondents spending a considerable amount of leisure time sedentary. The differences between the profiles, according to the eta^2^ value, were of medium effect size.

*Poor school experiences.* Not liking school and feeling high school pressure was concentrated among adolescents in the three profiles containing high psychosomatic complaints: (a) high psychosomatic complaints and low life satisfaction, (b) high psychosomatic complaints and self-rated poor health, and (c) high levels for all three cluster variables. The differences between the profiles were of large effect size for not liking school and medium for school pressure.

*Negative interpersonal relationships.* The same three profiles had particularly low scores for communicating with parents and receiving support from parents, teachers and classmates. The differences between the health profiles were mostly of medium effect size.

*Negative personal conditions.* Adolescents in these three profiles also stood out by reporting high levels of stress, loneliness, negative wellbeing and low self-efficacy. The differences between the health profiles were substantial, with large effect sizes, especially for experiencing loneliness.

The overall picture of the results is quite consistent, with adverse school, interpersonal and personal conditions concentrated among adolescents with health profiles that included several of the specific indicators of poor health, and where high psychosomatic problems were included in all these profiles.

The results of the study provide a consistent overall picture. Adverse school, interpersonal, and personal conditions were found to be prevalent among adolescents with health profiles characterized by high levels of psychosomatic complaints combined with high self-rated poor health and/or low life satisfaction.

Overall, young people who report only high levels of psychosomatic problems, without experiencing self-rated poor health or low life satisfaction, are not at risk of adverse physical, school, interpersonal and personal problems. Their scores on these types of problems are close to average. However, when psychosomatic problems are accompanied by self-rated poor health and/or low life satisfaction, these health profiles are stronger risk indicators. More than one in three persons with high levels of psychosomatic complaints in the profiles were persons with high levels of psychosomatic complaints only. Similar results are found for self-rated poor health. The adolescents who only self-rated poor health had slightly elevated scores for school, interpersonal and personal problems. However, the problems were considerably higher when self-rated poor health was accompanied by low life satisfaction and/or psychosomatic problems. These are the results of using a person-centered approach to identify different combinations of indicators of poor health.

We calculated how the seven health profiles differ for boys and girls separately on measures of problematic physical, school, academic, interpersonal, and personal conditions (not reported in tables). These analyses for both sexes show that the results for boys and girls, analyzed separately, were very similar to those for the total sample. Boys and girls in health profiles that included two or more of the specific indicators of poor health had worse adverse school, interpersonal, and personal problems than boys and girls in clusters with high levels of psychosomatic problems only or self-reported poor health only. Boys and girls in the cluster with high levels of all cluster variables reported significantly more adverse school, interpersonal, and personal problems than respondents in all other clusters.

## Discussion

The present study makes several contributions to the existing literature on adolescent mental health. A key question was whether the three indicators of poor health were similarly related to adverse physical, school, interpersonal, and personal conditions. This would be likely if the indicators were substantially related to each other and shared the same variance. We found that the three indicators of poor health formed a factor with factor loadings ranging from 0.66 to 0.78 across countries and survey years. It appears that the three health indicators share much of the same variance and we found support that they relate in similar ways to adverse physical, school, interpersonal, and personal conditions.

Despite high common variance, the person-centered (cluster) analysis shows that adolescents are characterized by seven distinct health profiles based on the three health indicators and that profile membership has implications for associations with the different adverse psychosocial conditions. Two of the profiles reflected good health and included most adolescents. The other five profiles captured different health problems. Here, some adolescents had health profiles characterized only by high psychosomatic complaints or only by self-rated poor health, while other profiles were characterized by two or all three indicators of poor health simultaneously. The present study suggests that the substantial correlations among common health indicators obscure the crucial point that none of these indicators, when occurring in isolation, indicates a high risk of adverse physical, school, interpersonal, and personal conditions. It is when several of these indicators occur simultaneously that the potential problems become apparent. It does not seem possible to understand the implications of the three indicators of poor health for young people’s physical, school, interpersonal, and personal conditions without knowing how these indicators are related at the individual level.

### Psychosomatic complaints

Psychosomatic complaints have been the primary candidate for mental health problems and have been linked to individual, social, economic, and cultural conditions (2). The person-centered approach used in the present study shows that of the five risk profiles, four included high levels of psychosomatic complaints. One of them was characterized by high psychosomatic complaints only. The other three also had high levels of self-rated poor health and/or low life satisfaction. While the various measures of school, interpersonal, and personal problems were at an average level for the first profile of adolescents with only high psychosomatic complaints, these problems were considerably higher for the latter three profiles. These very different findings should be seen in the context of the broader literature that has lumped these four profiles together in variable-centered analyses of the role of psychosomatic problems in their functioning at school, home, and leisure.

Adolescents with high levels of psychosomatic problems in addition to self-rated poor health and low life satisfaction, a total of about 6% of all adolescents in the five countries, is the essential risk group. On almost all of the measures we used to characterize the different health profiles, this group of adolescents stands out and differs significantly from the other profiles.

Adolescents with high levels of psychosomatic complaints appear to form a diverse group. Some of these individuals demonstrate personal, interpersonal and social adjustment problems almost indistinguishable from those of the average adolescent. In contrast, others exhibit significant disparities, displaying even more pronounced differences than any other cluster group. An explanation is necessary to facilitate comprehension.

A substantial body of research has demonstrated that the majority of adolescents self-report good health and high life satisfaction (22). However, daily life experiences may impact psychosocial functioning. Psychosomatic complaints have been shown to manifest as responses to everyday stressors resulting from the perception of unpredictable and uncontrollable life events (30). Our findings suggest that such reactions could influence adolescents’ perceptions of poor health and/or low life satisfaction, which could negatively impact their psychosocial adjustment. However, if these reactions do not affect adolescents’ broader conceptions of health and life satisfaction, they may not lead to more adverse psychosocial problems. This idea merits further exploration in future longitudinal studies of adolescent mental health.

### Self-rated poor health

The group of adolescents who reported high levels of self-rated poor health as their only high health indicator reported somewhat higher levels of school, interpersonal and personal problems than the average person. However, when high levels of poor health were combined with high levels of psychosomatic complaints, these problems were significantly higher.

Longitudinal studies have found that self-rated poor health is associated with future morbidity, mortality, and higher rates of medication use and health service utilization (12, 13, 15). In the present study, all three mental health profiles that included high levels of self-rated poor health differed significantly from the other mental health profiles in one respect. Adolescents in these profiles reported less frequent physical activity and more time spent in sedentary behaviors. This may have profound policy implications, as it is during the adolescent years that some adolescents who perceive themselves to be in poor health may develop and/or maintain sedentary lifestyles that may have negative consequences for their future health in many ways. In adult and older populations, perceived health status is widely recognized as a strong predictor of future mortality (31).

### Life satisfaction

In contrast to psychosomatic complaints and self-rated poor health, cluster analysis did not produce a health profile in which low life satisfaction was the only significant indicator of poor health. Low life satisfaction was only observed in association with low self-rated health and/or high rates of psychosomatic complaints. A minority of published longitudinal studies have used life satisfaction as an independent variable. However, results indicate that low life satisfaction may be a longitudinal risk factor for future adjustment problems. To illustrate, low life satisfaction has been identified as a risk factor for increased fear of missing out on social media (19), peer victimization on Facebook (20), and relational victimization (18). These findings suggest that low life satisfaction may contribute to the development of these negative outcomes. The relationship between childhood maltreatment and depressive symptoms (16) has also been shown to be associated with increased importance placed on life satisfaction and increased activity and successful enactment over time (21). In addition, life satisfaction has been identified as a predictor of social support seeking and problem solving (17). We envision future longitudinal studies that do not necessarily assume that life satisfaction is the result of adverse psychosocial conditions, but instead consider low life satisfaction as a potential risk factor for a range of personal, interpersonal, and social problems in adolescence. Low life satisfaction may ultimately be the critical indicator of poor health status, over and above psychosomatic complaints and self-rated poor health, that can explain the association between perceptions of one’s own problematic health in adolescence and future anxiety, depression, morbidity, mortality, medication use, and utilization of medical services.

### Implications for policy, practice, and research

The first implication of the results presented here is at the conceptual level. It is questionable whether it is appropriate to classify some health-related measures as indicators of good health per se (self-rated health and life satisfaction) and others (psychosomatic complaints) as indicators of poor health (28). The results can lead to uncertainty about which measures should be used to draw conclusions about the health status of young people, misunderstanding of trends over time, and, more generally, misinterpretation and misconclusions. For example, the Swedish Agency for Youth and Civil Society (32), a government agency responsible for monitoring the living conditions of young people, reported that 56% of young people aged 16–24 in Sweden experienced high levels of anxiety or worry in 2022. This was reported as a cause for concern. However, in the same study, 77% of the same young people rated their overall health as good. Therefore, a nuanced discussion is needed to combine information from different individual health-related measures.

In this context, both policy discussions and arguments (33, 34) and numerous empirical studies (35–37) have reported that increasing trends in psychosomatic complaints are evidence of an increase in mental health problems among young people in general in the Nordic countries. A notable omission in these policy discussions and empirical studies is the lack of consideration of the rather high stability of trends in self-rated health and life satisfaction from 2002 to 2022 in the Nordic countries (23, 38).

Regarding psychosomatic complaints, we have shown in the present study that young people with high levels of these complaints are not a homogeneous group. Nor are high scores necessarily a risk factor for various negative individual and societal outcomes. Adolescents whose mental health profile showed only elevated levels of psychosomatic problems (and not self-rated poor health or low life satisfaction) included over one in three adolescents with elevated levels of psychosomatic complaints in the cluster analysis, but had a risk profile for adverse physical, school, interpersonal and personal conditions that did not distinguish them from the average person.

Finally, in terms of implications for research, person-centered research may be able to provide more nuanced information about the specific conditions that affect adolescents’ health status (27) than variable-centered analyses.

### Strengths and limitations

A strength is the use of a large dataset from the international HBSC study, which is now being conducted according to a common research protocol in 50 participating countries in Europe, Central Asia and Canada. The protocol covers all steps of data collection, including validation and translation of instruments, questionnaire design and piloting, sampling of schools and students, and cleaning and coding of collected data.

The main strength of the present study is the combination of variable-centered and person-centered analyses (25), where the latter type of analyses makes it possible to examine adolescent groups in terms of their distinctive health profiles and to identify those profiles that are associated with an increased risk of adverse physical, academic, interpersonal, and personal outcomes. Psychosomatic complaints has been the primary health measure in previous studies when associated with aversive psychosocial conditions. Our contribution to the literature is to show that health profiles with high levels of psychosomatic complaints are not necessarily associated with high levels of psychosocial problems. This is only true when high psychosomatic problems are combined with self-rated poor health and/or low life satisfaction.

The study lacks information from independent sources—parents, teachers, and friends—that would have made it possible to determine whether other people recognize the problems of the young people in the cluster groups characterized by high levels of several of the self-reported indicators of poor health. In addition, the study lacks information on the causal relationships between the three indicators of poor health, as well as the causal relationships between the indicators of poor health and the different types of adverse psychosocial conditions.

## Conclusion

Psychosomatic complaints are often used as a proxy for mental health problems. However, the presence of high psychosomatic problems exclusively does not imply that these adolescents are exposed to (or affected by) more adverse psychosocial conditions than other adolescents. First, variable-centered analyses show that the associations between psychosomatic complaints and adverse psychosocial conditions are similar to those found for self-rated poor health and low life satisfaction in relation to these adverse psychosocial conditions. Thus, psychosomatic complaints do not emerge as a primary marker of exposure to adverse psychosocial conditions. Second, person-centered analyses show that the association between adverse psychosocial conditions and psychosomatic problems is contingent on the perception of poor health and low life satisfaction among adolescents with psychosomatic complaints. That is, the association depends on the overall health profile rather than on a single health indicator. Thus, the person-centered approach seems to facilitate better discussion and targeted implementation of interventions and policies.

## Data Availability

The original contributions presented in the study are included in the article, further inquiries can be directed to the corresponding author.
